# Evidence of the impact of CLN2 and CLN3 Batten disease on families in the United Kingdom

**DOI:** 10.1186/s13023-025-03747-8

**Published:** 2025-05-12

**Authors:** Sara E. Mole, Paul Gissen, Shannon Nordstrom, Suzanne Wait, Louise Allen, Mathilda Antonini, Liz Brownnutt, Richard Brown, Barbara Cole, Frances Gibbon, Robert H. Henderson, Sarah Kenrick, Zlatko Sisic, Bob Thompson, Joanna Nightingale

**Affiliations:** 1 UCL Great Ormond Street Institute of Child Health, 30 Guilford St, London, WC1N 1EH UK; 2 NIHR Great Ormond Street Hospital Biomedical Research Centre, Great Ormond St, London, WC1N 3JH UK; 3 The Health Policy Partnership, 68–69 St Martin’s Lane, London, WC2N 4JS UK; 4 Cambridge University Hospitals, Hills Rd, Cambridge, CB2 0QQ UK; 5 Great Ormond Street Hospital for Children (GOSH), Great Ormond St, London, WC1N 3JH UK; 6 The Batten Disease Family Association (BDFA), PO Box 379, Shipley, BD18 9GE UK; 7 Addenbrookes Hospital, Hills Rd, Cambridge, CB2 0QQ UK; 8 Noah’s Ark Children’s Hospital for Wales, University Hospital of Wales, Heath Park Way, Cardiff, CF14 4XW UK; 9 Moorfields Eye Hospital, 162 City Rd, London, EC1V 2PD UK

**Keywords:** Batten disease, Neuronal ceroid lipofuscinoses, CLN2 disease, CLN3 disease, Parents, Siblings, Family experiences

## Abstract

**Background:**

Neuronal Ceroid Lipofuscinoses (NCLs), also known as Batten disease, are a group of inherited neurodegenerative disorders that mostly arise in childhood. Each of the NCLs is a genetically distinct disease caused by variants in at least 13 different genes (*CLN1–CLN14*). NCLs are neurodegenerative, and symptoms can include a combination of childhood dementia, epileptic seizures, motor decline and vision loss, and eventually lead to premature death. There is currently no cure for any subtype of NCL, however, enzyme replacement therapy is available for CLN2 disease, and several treatment strategies are being explored for other NCL subtypes. Early diagnosis and initiation of supportive services (e.g. health, education, social services) are essential to preserve quality of life. Only a few studies have investigated family experiences with NCL, many of which are international in scope.

**Methods:**

A mixed-method research study was conducted in the UK to understand family experiences in CLN2 and CLN3 disease. It involved an initial literature review, followed by in-depth qualitative interviews. Interview data were analysed using a thematic analysis. Thirteen families (*n* = 13) participated in the interviews. This represented 16 parents (11 mothers and 5 fathers) of 18 children (10 diagnosed with CLN3 disease and 8 diagnosed with CLN2 disease). Findings were analysed jointly across CLN2 and CLN3 disease.

**Results:**

Six overarching themes emerged from the analysis: difficulty in recognising early symptoms; the shock of a diagnosis; the demands of caring for complex and ever-changing needs; a constant battle to access appropriate and timely support services; the extensive impact on the unaffected sibling; and the all-encompassing impact on the family.

**Conclusions:**

This study contributes novel UK specific data on family experiences and unmet needs in CLN2 and CLN3 disease. More needs to be done to ensure NCLs are diagnosed early, and timely local support services are made available to protect quality of life for both the affected children and their families.

**Supplementary Information:**

The online version contains supplementary material available at 10.1186/s13023-025-03747-8.

## Background

Neuronal Ceroid Lipofuscinoses (NCLs), also known as Batten disease, are a group of inherited neurodegenerative disorders that usually appear in childhood [1–4]. Pathologically, they are characterised by the accumulation of autofluorescent storage material in the lysosomes [1]. Each of the NCLs is a genetically distinct disease, caused by variants in at least 13 different genes (*CLN1–CLN14*) [2]. The most common subtype of NCL in the UK are CLN2 and CLN3 disease [5]. 

The Batten Disease Family Association (BDFA) estimates that there are 100 to 150 children, young people and adults currently living with a NCL diagnosis in the UK [6]. Of these, there are between 30 and 50 children with CLN2 disease, and between 30 and 40 children and young people with CLN3 disease [7, 8]. The remaining cases are a combination of other genetic types [4, 6]. Because most forms of NCLs are inherited as autosomal recessive, families can have more than one child with NCL [1, 4, 9]. 

The NCLs cause a combination of symptoms that can include childhood dementia, seizures, motor decline and vision loss [2, 10]. Eventually, the disease leads to premature death. All types of NCLs share similar clinical features but vary in age of onset, symptoms and rate of progression [10, 11]. There is currently no cure for any subtype of NCL, and treatment is mostly palliative [12]. However, enzyme replacement therapy (cerliponase alfa) is available that slows CLN2 disease progression and improves quality of life; and several treatment strategies are being explored for other NCL subtypes [13–15]. Cerliponase alfa was licensed for use by the European Medicines Agency in 2017 and was made available by NHS England in 2019, under a five-year managed access agreement (MAA) that is under evaluation for extension [13, 15]. 

If left untreated, classic late infantile CLN2 disease typically presents between the ages of 2 and 4 years and manifests as new onset seizures and language delay or deficits [16, 17]. Motor dysfunctions also occur in the early stage of disease, manifesting as clumsiness or ataxia [16]. From 3 to 6 years of age there is rapid progression in almost all children and a decline in motor and language skills, and vision and cognitive function– alongside worsening seizures and involuntary muscle jerks [16]. Children are typically severely impaired and dependent on their carers by 6 years old, becoming wheelchair bound and non-ambulant, with many needing a feeding tube [9, 16, 18]. Death usually occurs between the ages of 6 and 12 years old [9, 16]. Atypical phenotypes of CLN2 disease can vary in age of onset, rate of progression, disease manifestation and death [4, 9]. 

Classic juvenile CLN3 disease is slowly progressive, sometimes with 20 or more years between symptom onset and death [19]. While the evolution of the disease is characteristic and predictable in relation to the order of symptoms, there is significant variation among children and young adults in the time it takes to develop the next symptom, and not all children will experience every symptom [20]. The most common first symptom is rapid vision loss between the ages of 4 and 7 years [11, 21]. Children usually go from having normal vision to functional blindness in months [11]. Cognitive dysfunction (e.g. difficulty with concentrating, learning at primary school, short-term memory) manifests from 7 to 10 years, with behavioural symptoms (e.g. aggression, sleep disturbances) from 8 to 10 years [11, 21]. Seizures occur between the ages of 10 and 12 years [11, 22]. Later in the disease course, children lose ambulation and become wheelchair users by late adolescence, and may develop cardiac arrythmias and feeding difficulties [19, 21]. Psychosis, hallucinations and/or childhood dementia can appear at any point during CLN3 disease [20]. Children and adults affected by CLN3 die between the ages of 15 and 35 [7]. There also exist later onset and more slowly progressing forms of CLN3 disease [4]. 

A number of studies have contributed to our understanding of the epidemiology, genetics, natural history and management of NCLs [22–26]. However, only a few studies have investigated family experiences with CLN2 disease and CLN3 disease, including the impact on siblings of children with the condition. Notable studies include that by Schulz et al. (2020) which investigated family experiences with CLN2 disease in the UK and Germany and found that families cope with difficult emotional, physical, professional and financial challenges [27]. Another study by Krantz et al. (2022), investigating parental experiences of having a child with CLN3 disease in Sweden, detailed the significant disruption to the family unit [28]. A UK-wide study by Malcolm et al. (2014) of the impact on siblings of children with NCL found that the siblings experience a range of complex emotions such as daily anxieties, worry and sadness due to concerns about their brother or sister’s health [29]. A recent international study on family experiences in CLN3 disease by Schulz *et al. (*2024), which included the UK, found that the disease had a financial impact on 81% of families due to the parents’ reduced ability to work and increased health-related expenses, and caused marital strain in 46.5% of families [30]. Lastly, Cozart et al. (2017) examined marital quality among NCL parents and found that their marital quality was significantly lower than that reported in studies of the general population, or of parents of children with chronic, but non-neurodegenerative disease [31]. 

Despite these impactful studies, there remains a need for more detailed UK specific data on families affected by NCLs and the overall impact of the disease on the family unit. This study was initiated to fill this evidence gap, and to provide evidence to support calls for policy and practice change that could address families’ unmet needs in the UK.

For reasons of feasibility, the study focused on families affected by CLN2 and CLN3 disease only, the most common NCL subtypes in the UK [5]. Findings will be used to guide the policy advocacy work of the BDFA in years to come, and may also be helpful to inform work in other countries.

## Methods

A mixed-methods research study was conducted in the UK between March 2022 and November 2023. It involved an initial literature review to gather all existing evidence of family experiences with CLN2 and CLN3 disease, followed by in-depth qualitative interviews with families affected by CLN2 and CLN3 disease. Interview data were analysed using a thematic analysis, and findings were validated by the study participants. The analysis focused on finding common themes that affected all study families, with a view to gaining a better understanding of how having a child with CLN2 and CLN3 disease affects the family unit and what potential changes may be needed to best address their complex needs. Findings were combined across CLN2 and CLN3 disease.

### Literature review

A structured, non-systematic literature review was carried out in March 2022 to gather all English-language international literature on the burden of NCLs on families, treatment and care, policy developments and family experiences. Published literature was identified on PubMed using a search strategy. Of 1,416 articles identified, 362 were reviewed and 101 retained in the findings report. Additional literature was found using the snowball sampling method. Findings from the literature review informed the creation of the qualitative interview guide to ensure it was focused on the most relevant areas for families affected by Batten disease.

### Qualitative interviews

#### Recruitment of participants

For feasibility reasons, a mix of voluntary responses and purposive sampling was used to recruit families for this study. Families of children with CLN2 or CLN3 disease were contacted and recruited via the BDFA database, which includes nearly all families affected by NCL in the UK (approximately 110 families in total). Families who expressed an interest in the study were asked to complete an application form, and families were selected based on the characteristics of interest (Table 1). Families volunteered to take part in the study and, as a gesture of thanks, were provided with a gift card.

**Table 1:** Characteristics to guide family selection

Selection criteria	Exclusion criteria
• Families of children with a confirmed diagnosis of CLN2/3 disease	• All other types of NCL
• Parents or adult siblings who currently care, or had previously cared, for a child or children with CLN2/3 disease	• Members of the extended family who currently care, or had previously cared, for a child or children with CLN2/3 disease
• Both atypical and typical CLN2/3 disease	
• All ages and ethnicities	
• All UK nations (Wales, England, Scotland and Northern Ireland)	
• Received or receiving cerliponase alfa; and did not receive cerliponase alfa (CLN2 only) • Any number of children in the family with CLN2/3 disease	
• Any number of children in the family with CLN2/3 disease	

#### In-depth interviews

An interview guide was informed by findings from the literature review. It included 12 questions (most of which were open-ended) covering the three focus areas of investigation: early symptom onset and diagnosis, support and care, and the impact of the disease on the family unit (Appendix A). The interview guide was pilot tested with two families (one with CLN2 disease and one with CLN3 disease) and revised before conducting the remaining family interviews. To capture the family experience related to having additional children in the family diagnosed with the condition, the guide was adapted slightly for families who had more than one child affected by NCL.

Qualitative in-depth interviews were carried out virtually on a video call (Zoom) between April and June 2023. They were led by an experienced researcher and were scheduled for 60 min. Interviews were video recorded, transcribed verbatim and anonymised to remove any personally identifiable information. The study was conducted in line with the 32-item COREQ framework for qualitative research [32]. 

#### Participant involvement

Consideration was given to fully involve and inform study participants through all phases of the study. An introductory meeting was held with all families interested in participating in the study, with all pertinent information summarised in an information sheet and discussed verbally with families during the meeting. Informed consent was secured from participants prior to the in-depth interviews, and the interview guide was shared with families ahead of their interviews. Interview transcripts were shared with families following the interview to check for accuracy and allow participants to make any corrections. A study meeting was held with families to present research findings and gain their feedback. Ahead of the meeting, study findings were shared with all families via email. Four parents (*n* = 4 of 16) attended the study meeting where research findings were presented, discussed and adapted.

### Data analysis

A thematic analysis was conducted on all interview transcripts to identify themes and patterns in the data gathered from the families affected by either CLN2 or CLN3 disease. Transcripts were read several times before coding the interview data by author SN using NVivo, a thematic analysis software program. Theme generation followed Braun and Clarke’s six stages for thematic analysis using an inductive approach, meaning themes were derived from the interview data rather than pre-determined based on existing research [33]. An inductive approach was deemed most appropriate given the limited research available on Batten disease family experiences, and to adhere to our wish to capture the families’ perspective.

Data were analysed on a semantic level with a realist approach; the themes were identified within the explicit or surface meanings of the data without looking beyond what a participant has said or what has been written [33]. The coding was discussed twice between authors SN and SW, and subsequently revised before the coding tree was finalised (Appendix B). Preliminary themes were created by grouping codes together and then discussed by authors SN, SW, JN, LB, and ZS until an agreement was reached. Themes were then validated by all authors and study participants before being finalised.

## Results

Thirteen families (*n* = 13) participated in the interviews. This represented 16 parents (11 mothers and 5 fathers) of 18 children (10 diagnosed with CLN3 disease and 8 diagnosed with CLN2 disease). Of the 18 children, 45% were female (*n* = 8) and 55% were male (*n* = 10); 33% were actively receiving cerliponase alfa (*n* = 6) at the time of the study application and 11% had received cerliponase alfa in the past but had since stopped (*n* = 2). The characteristics of the 18 children affected by CLN2/3 disease are provided in Table 2.

**Table 2:** Characteristics of children with CLN2 or CLN3 disease

Characteristics	CLN2 disease (*n* = 8)**		CLN3 disease (*n* = 10)	
	Median	Range	Median	Range
Current age (years)*	7.5	4–16	10	7–17
Year of diagnosis	2021	2015–2022	2017	2016–2022
Age at diagnosis (years)	4.5	2–13	7	2–11

*At the time of application

**Two children with CLN2 disease had an atypical clinical phenotype

Two families (*n* = 2) invited to participate in the study did not show up for the scheduled interview (one did not cite a reason, while the other cited care commitments); and another family (*n* = 1) withdrew from the study prior to the interview being scheduled (no reason cited). One family was scheduled for a second additional interview as the first one ran out of time to complete in full.

**Fig. 1:**
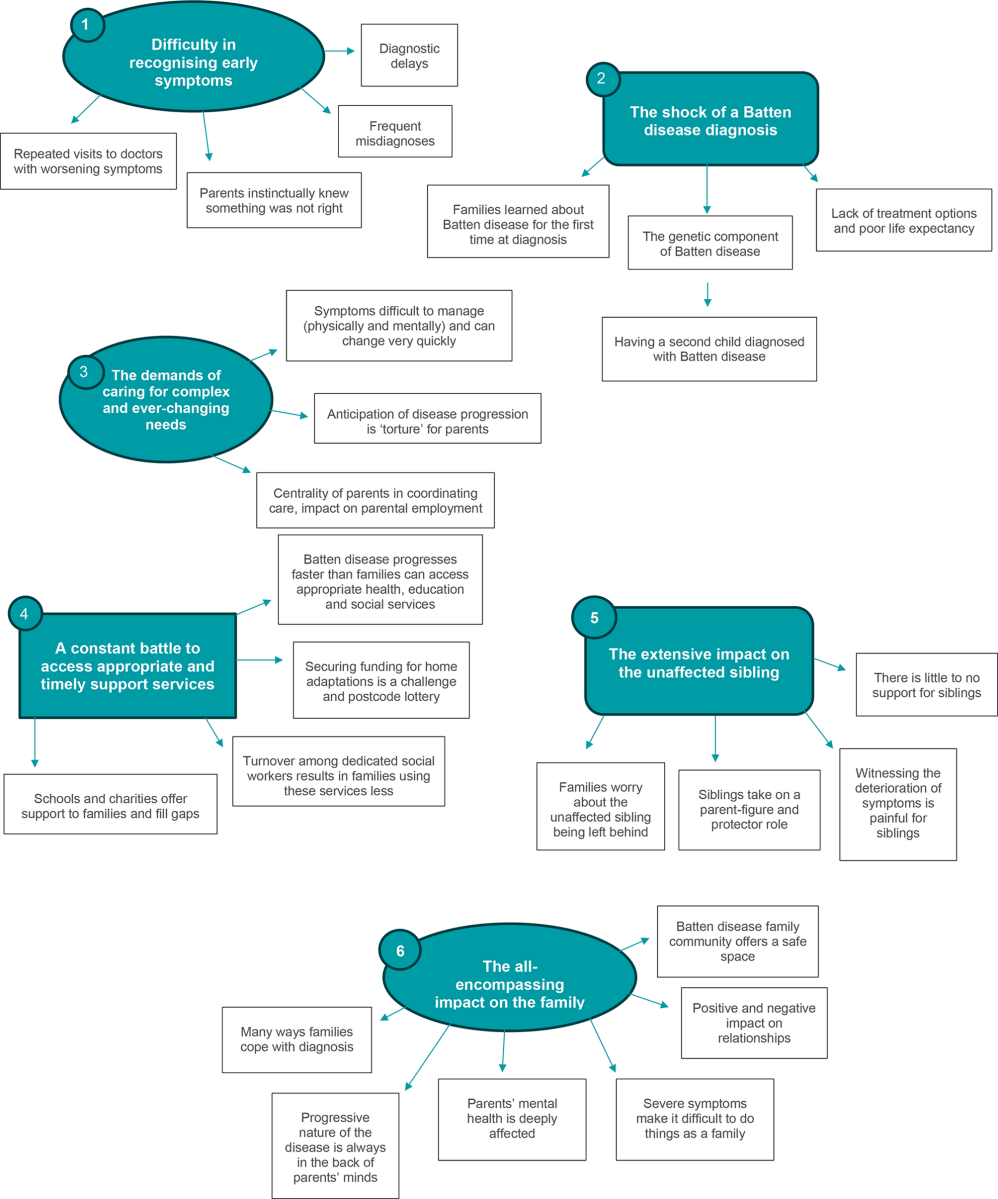
Map of six themes from thematic analysis

Most interviews were conducted in a one-on-one setting between the researcher (SN) and a parent (*n* = 10); three family interviews were conducted with two parents present (*n* = 3). In nearly all cases, parents took the interview in their family home (*n* = 11); one parent took the interview from a car (*n* = 1) and another in a public setting (*n* = 1). The children affected by Batten disease in the family were not present for the interviews. The thematic analysis resulted in six main themes (Fig. 1):

### Difficulty in recognising early symptoms

Although presentation of symptoms occurs at different ages for CLN2 and CLN3 disease, families’ experiences with diagnosis followed similar patterns. Initial symptoms of CLN2/3 disease were often subtle, and parents expressed that they instinctually knew something was wrong. Families recounted behavioural changes in both CLN2 and CLN3 disease mirroring attention deficit hyperactivity disorder (ADHD) or autism. Families impacted by CLN2 disease recounted early symptoms such as speech delays, learning difficulties, mobility deterioration, sensory issues and clumsiness, myoclonic jerks, and seizures. Early symptoms unique to CLN3 disease reported were speech delay, squinting, and eyesight deterioration. When parents brought these concerns to healthcare professionals, symptoms were often dismissed. Parents felt paranoid and frustrated as a result.

Every time I went to any doctor, I’m like, I know I sound crazy. But [child name] is different, like there’s something that’s not right, something that I don’t know, I could see a change. And I just felt crazy. Because the doctors looked at me like, What do you mean [about the symptoms]?. (CLN2)

This led families to a cyclical pattern of visiting doctors, optometrists or ophthalmologists (CLN3 disease), or having frequent hospitalisations for seizures (CLN2 disease), only to be told nothing was wrong, then returning weeks later with worsening symptoms. Families expressed that they were constantly pushing and demanding to have their concerns heard. One child affected by CLN2 had worsening seizures for over a year before epilepsy medication was started, and only when that failed was genetic testing triggered.

When the seizures started, it was so stressful. The seizures would be happening, we phoned the ambulance, and three different times we had to stay in the hospital for observation. (CLN2)

It was common for children to be misdiagnosed with a condition other than CLN2 or CLN3 disease. Initial diagnoses included macular degeneration, epilepsy, white matter disease, retinal dystrophy, retinitis pigmentosa, cone dystrophy and corneal dystrophy, among others. This led to families experiencing repeated emotional distress where they would come to terms with the initial diagnosis, then had to re-adapt to a new diagnosis.

We were devastated when we just thought it was retinal dystrophy. We thought like– wow, our lives are going to be a lot different. And the future that we envisioned for our child is going to look a lot different. It took a long time to come to terms with that.’ (CLN3).

This reality resulted in families experiencing diagnostic delays, meaning that families also experienced delays in accessing support services (Table 3).

If they had tested from the word go, it won’t change any of the outcomes, but we would have been able to put support in place for our child a lot sooner, because we would have known that our child’s eyesight was going to deteriorate fast. Regarding education and support, it wasn’t put in place fast enough. [By] the time they put things in place, [child’s name] eyesight had already deteriorated further. (CLN3)

**Table 3:** Time to diagnosis from symptom onset, for the first child in the family diagnosed with CLN2 or CLN3 disease

Type of NCL	Average (years)	Range (years)
CLN2 disease (*n* = 6)	2.05	0.33-8
CLN3 disease (*n* = 8)	1.55	0.42-6

### Shock of a diagnosis

Families received a diagnosis of CLN2 or CLN3 disease as a result of genetic testing to confirm the cause of worsening seizures (CLN2) or vision deterioration (CLN3). Families were often not explicitly informed that their child was being tested for NCL and felt shocked when the diagnosis was given, as they had never heard of NCL before. Only two families were told that NCL was being tested for, upon initiation of genetic testing. Another family paid privately to access hospital records to find out what diseases were being tested.

The doctor wouldn’t tell us what was being tested for, and we disagreed with that; we think [the doctor] should have told us. We paid privately to get our child’s hospital records to see what was being tested for. And obviously, one of those things was Batten disease. (CLN2)

Parents were further shocked by the poor life expectancy and lack of curative treatment options for CLN2 and CLN3 disease.

The general life expectancy, it’s not fair. Like you try for kids, then you find out they have a rare disease [touching hand to face in disbelief] without treatment, without a cure, it was a disaster. (CLN2)

The genetic component of CLN2 and CLN3 disease was an additional shock to an already devastating diagnosis. Two families expressed complex emotions such as guilt and regret for not having known about the genetic risk of NCL before having children.

My child has suffered [Batten disease] because me and my wife never knew that we have a genetic problem. There should be more awareness so that when you marry or when you plan to have children, you can get your genetic tests done so you don’t get this pain after you have children. (CLN3)

Additionally, parents with multiple children were faced with the difficult decision of whether to test their other children for CLN2 or CLN3 disease. One parent mentioned having an empathetic genetic counsellor made a positive difference during this difficult time.

We decided to get the other children tested. We weren’t going to because we were still trying to get our heads around the fact that [child’s name] had it. And we didn’t really want another hammer blow. We decided that it’s better to know. (CLN3)

Five families endured the pain of having two children diagnosed with CLN2 or CLN3 disease, owing to the genetic nature of the condition. The second diagnosis was often in a younger sibling who had not yet displayed symptoms of the disease. The diagnosis happened quickly for the second child in the family, usually within a few weeks or months of the first familial diagnosis.

### The demands of caring for complex and ever-changing needs

Tending to the complex and ever-changing needs of a child with CLN2 and CLN3 disease can be all-consuming, both physically and mentally– leaving families feeling exhausted and drained. Symptoms can change quickly and families feel they have to constantly be ten steps ahead. Symptoms also present differently depending on the child, adding to the complexity of caring for them.

It just drains you. I think you just get tired, but not like where you get a good night’s sleep and you’re better. You’re just tired in every aspect and no matter what you do, you just can’t, you just can’t stop feeling tired. (CLN2)

Witnessing the deterioration of the child’s abilities due to symptom progression, coupled with the uncertainty of not knowing what the future holds and how the disease is going to develop further, was described by one family as ‘torture’. Families described having constant worries and fears about the future. One family described feeling as though every day is like a ticking time bomb.

Right now, fortunately, it’s just sight loss. We’re not dealing with neurological signs at this point. But it’s just…when your mind starts to get ahead of yourself…the future and what that will look like– it, you know, gets quite upsetting. (CLN3)

Parents play a central coordinator role in managing their child’s care– acting as the fount of all information related to their child’s condition and often even managing communications between healthcare professionals and hospital departments.

I’ve [had] to pass messages on between professionals because the lack of communication is just ridiculous. That’s one of my big bugbears at the minute. (CLN2)

The increased caregiving responsibility has an impact on parental employment to varying degrees. Three parents had to step back from full-time employment permanently - one sold their business - owing to their caregiving responsibilities and the time involved. An additional two additional parents took temporary leave or sabbatical to help process the diagnosis and care for their child. Lastly, a further two parents expressed that their flexible working situations made all the difference to caring for their child and attending frequent medical appointments.

I used to work full time and had my own business. But I had to sell everything… Because [child’s name] needed attention 24 hours due to losing their eyesight very quickly. (CLN3)

### A battle to access appropriate and timely support services

CLN2 and CLN3 disease progresses faster than families can access appropriate social, health and educational support services. Once families gain access to the desired support service, the disease has often progressed, and they may need access to something different. Accessing the needed services has been described as a constant fight, a battle and waiting game due to long waiting times and complicated eligibility requirements.

Every day that we’re fighting is a day less that we get with our children. We don’t have a lot of time anyway, and we’re often wasting these days [trying to access support] where we should be enjoying [time with their children]. (CLN2)

Social workers are a great help to families in coordinating access to social services, with some families noting their social worker has become fully integrated into their family. However, for the majority of families, staff turnover and inconsistencies among social workers created additional stress and resulted in the families using these services less often.

If you’ve got children that have dementia, the last thing you want to do is keep introducing them to new social workers who are supposed to be the people that they’re supposed to, you know, confide in and talk to. (CLN3)

Securing funding for home adaptations is a challenge for many families. To support a child with complex needs, homes require costly equipment such as hoists and stairlifts, along with wet rooms, space that is wheelchair accessible, and other modifications. Often, this is partly funded by Disabled Facilities Grants (DFG), but it has been described as a postcode lottery and insufficient to cover all the costs needed to adapt the home. Families had different experiences in accessing DFG. When it was not available, or did not cover the full cost, families had to fundraise or use their savings to pay privately, resulting in an enormous financial burden.

The money we’re getting from the local authority doesn’t scratch the surface. We’re having to do a lot of fundraising and pulling apart our savings [to renovate our home]. (CLN3)

Anticipatory education provision to allow children to maintain skills and quality of life– particularly in later stages of disease progression– is critical for children affected by CLN2 and CLN3 disease due to complex symptoms that can adversely impact effective learning [34]. Anticipatory education provision was often implemented too slowly. Important educational support needed to accommodate children with complex needs– such as specialised school placements or putting in place an Education Health Care Plan (EHCP)– was often accompanied by long waiting times.

We knew [our child] was sight impaired in January of 2022. They [the mainstream school] did the best they could to help [child]. And that’s when they started the application for the education health care plan. And it didn’t get approved, I think, until January 2023. (CLN3)

School transitions were often challenging for the child and families, but once children were enrolled in specialised schools, or had access to EHCP, families expressed that it made all the difference. School teams went above and beyond to accommodate their children and were always four steps ahead of the family needs. Specialised schools in particular offered everything under one roof, building a support system around the child (such as a variety of allied healthcare professionals) and making various appointments easier to manage.

[The specialised school] covers everything. They’ve got a pool. They’ve got the nurses. They’ve got the OTs [occupational therapists]. They’ve got the physios. They’ve got a dentist. They’ve got a lot of people in there. And for me that has been a big help knowing that everything’s in one place. (CLN3)

Schools and charities play an important supportive role to families, providing peer-to-peer support, financial support (e.g. grants) and psychological support. These organisations also offer respite hours as well as helping families navigate the educational, health and social services they are entitled to, guiding them to the right resources and in many cases applying for support on the family’s behalf.

### The extensive impact on the unaffected sibling

Families expressed worry about the unaffected siblings being left behind. They were acutely aware of their attention being focused on caring for their child with CLN2 or CLN3 disease and ensuring that siblings do not feel forgotten. Families felt that siblings do not get enough one-on-one time with parents, so the parents often consciously carved out alone time with the unaffected sibling. Parents tried to protect and maintain a sense of normality for siblings, despite these challenges.

And you know, when you know your child has this problem, all your attention goes to them. But at the same time, we try to give [the siblings] the same sort of feeling that they do not feel left behind or anything. (CLN3)

Siblings can become very independent and often look after their own needs, such as morning and bedtime routines. Siblings take on a carer role for their sibling with the disease without being asked, saying it is intuitive and as if they have become a third parent. Siblings can also become fiercely protective of their affected sibling, defending them in social situations with peers. To the contrary, one parent expressed that the unaffected sibling distanced themselves from the family, preferring to engage in school and social life, and does not wish to discuss the disease.

[The sibling] threw himself into learning and social life, and kind of shut the door on us. We tried counselling, it just wasn’t for [the sibling]. We tried sibling support groups, but [the sibling] always felt that he was too old for the groups. We looked at getting registered as a young carer. [The sibling] just didn’t want to. We were doing fundraising and awareness. [The sibling] doesn’t want to be involved. (CLN3)

As the disease progresses, the worsening of symptoms can be incredibly hard for unaffected siblings to witness. This has been described by siblings as a loss, and can lead to complicated feelings of guilt over the fact that they are healthy while their sibling is not. Parents describe the difficulty in broaching this topic with siblings; they want to protect them but, equally, feel the need to explain the reality of the disease when more questions begin to arise. Older siblings tend to be affected differently compared to younger siblings, for whom it is all they have known.

The last couple of years have taken a real hit on [the unaffected sibling’s] mental health and trying to navigate between his world and [the affected sibling’s] world, because [the affected sibling] was quite stable for a long time. But in the last 18 months, that’s when [the affected sibling’s] vision started deteriorating. We noticed that [the unaffected sibling’s] behaviour changed at the same time that was happening. I think that’s probably because [the unaffected sibling] is the eldest and feels responsibility. And one thing that [the unaffected sibling] has said in the past when they were younger was, “Why is it [the affected sibling] and not me?” (CLN2)

Parents have found that there is little support available for siblings. One family said that siblings ‘don’t exist’ in the eyes of support services. Siblings need readily accessible local support– such as counselling or sibling support groups– to provide a safe space to discuss their feelings and emotions. Sibling respite hours would be beneficial, along with groups that can organise activities for siblings to get out of the house. Families might not have the capacity to spend time getting the unaffected sibling out of the house because of caring responsibilities.

You have to look at [support services] as a family. The unaffected children, it’s as life-changing a diagnosis for them as it is for everybody else. (CLN3)

### The all-encompassing impact on the family

Families coped in many ways; they had difficulty accepting the diagnosis but were dealing with it as best they could. Families either took comfort in gathering as much information as possible on the disease, while others found it easier to take things day by day.

I do think about the future, and know what’s going to come, but I live day to day. And if it’s a good day, it’s a good day. And if it’s a bad day, it’s a bad day, but I don’t…how do I put it.,.I just try and live as a normal family [if] possible. Like, just don’t try and think about it, to be honest. (CLN2)

One family said that the fact that the disease is a progressive and terminal illness is always at the back of their minds, and with that comes the worry of one day having to say goodbye to their children. Four families expressed the importance of creating positive memories for their children and giving them the best life possible in the time that they do have; two of which have done extensive memory-making trips to gain as many experiences for their children as possible.

When we heard about [Batten disease], we started reading that, you know, one day we will have to say goodbye to our children. They will be only teenagers. (CLN2)

Having a child with CLN2 or CLN3 disease has a big impact on parents’ mental health. Two parents revealed that they had to take time off work as sick leave due to having had a mental breakdown or difficulties with mental health. Families described the importance of having mental health support, but also the difficulty they have had in accessing it through the NHS. One parent was told they were not eligible because they didn’t hit the right scores for NHS counselling.

Well, since he’s been diagnosed, it has changed our life completely. In different ways. One is that, emotionally, he has just broken us. I mean, if I give you an example to explain this, my sister was saying to me that it’s been two, three years, I’ve never seen you smile. Every little thing makes me emotional; it has made me very sensitive. (CLN3)

Severe symptoms of CLN2 and CLN3 disease make it difficult to do things as a family. Where symptoms are severe, families miss out on activities and holidays together owing to a lack of accessibility and difficulty in coordinating outings– especially if there are multiple siblings. Families tend to split up and do things separately.

[Child’s name] typically has 40 to 50 absent seizures an hour. It’s hard to do something with all that going on, but we still somehow manage to get through. (CLN2)

The disease can have both a positive and a negative impact on personal relationships. Parents either turned inwards and preferred to rely on themselves, not wanting to burden friends or family by asking for help. While others experienced a coming together of family and friends, forming a source of support upon which the family can rely on both emotionally and practically. Extended family members (especially grandparents) supported with caring for the child with CLN2 or CLN3 disease, allowing the parents time to rest. Three families expressed they had friends fade away or back off, which left them feeling confused and hurt.

The Batten disease family community offers a place of comfort and support. Families described the community as a ‘safe space’ where they felt solace in having a network of families going through the same thing. Connecting with other families provides a valuable opportunity to ask questions and seek advice. The importance of the annual BDFA family conference and virtual family support networks came through strongly in all family interviews. One family described these forums as an important part of their journey towards acceptance of NCL.

There’s something really powerful about looking into the eyes of another parent that knows exactly all of those feelings, all of those scenarios. I have, like, the best friends that I love with every part of me and who love [our children] as much as I do. But there’s something different when you look at another mum, and she has a child with Batten disease as well. It’s something you can’t put into words. (CLN3)

## Discussion

To our knowledge, this is the largest recent study to be conducted in the UK investigating family experiences with CLN2 and CLN3 disease. The study took place at a point in time when, following COVID-19, UK health and care services are facing challenges on several fronts, including record waiting lists for GP and hospital appointments, staff shortages, and many people struggling to access care. At the same time, public satisfaction with the NHS is at a four-decade low [35]. 

The findings demonstrate that children affected by CLN2 and CLN3 disease continue to face diagnostic delays. Families have difficulty accessing appropriate and timely local support services (e.g. health, social and education) and therefore have high unmet needs. The impact on the family unit is all-encompassing, including on the siblings of children with the disease, who are often poorly served by support services. Importantly, this study adds a new dimension to existing research by examining family experiences with early symptoms of CLN2 and CLN3 disease and by uncovering the diagnostic odyssey of families.

Given the degenerative nature of Batten disease, early diagnosis is vital to ensure that children and their families can be directed in a timely manner to the cascade of support services that will help protect their quality of life, including financial support, psychological support, local or national peer-support groups, sibling support and specialist education [22]. Early diagnosis also allows children with Batten disease to be guided to specialist-led care, which is needed to provide them with optimal treatment approaches to help manage symptoms and access any relevant clinical trials [7, 22, 24]. In CLN2 disease, early diagnosis enables children to be evaluated for their suitability for the only existing disease-specific treatment for NCLs– enzyme replacement therapy, cerliponase alfa, which can slow disease progression but is not curative [14, 15]. 

Taking a pre-emptive approach to early diagnosis can have a significant impact on children and their families. Yet our study found that children affected by CLN2 and CLN3 disease continue to experience delays to diagnosis– despite the availability of cerliponase alfa during the last five years in the UK. Of the children who received the first diagnosis of CLN2 or CLN3 disease in their family, the average time to diagnosis was 1.55 years for CLN3 disease (ranging from 0.42 to 6 years; *n* = 8) and 2.05 years for CLN2 disease (ranging from 0.33 to 8 years; *n* = 6). These results are similar to previous international studies, which demonstrated that children with CLN2 disease take on average 2 to 3 years from symptom onset to be diagnosed, and a UK study which found it can take 1.5 to 5 years from initial disease onset for children to receive a diagnosis of CLN3 disease [22, 23]. In this study, the average delay to diagnosis for CLN2 disease (2.05 years) was longer than for CLN3 disease (1.55 years) which is not typical given that CLN2 disease progresses more rapidly. However, our study had one child with an atypical CLN2 phenotype, that took 8-years to receive a diagnosis from symptom onset, contributing to this finding. When re-calculating the average time to diagnosis for CLN2 disease, removing the child who had an atypical phenotype from the calculation, the average time to diagnosis was significantly shorter at 0.866 years.

In many cases, subsequent children in a family previously diagnosed with CLN2 or CLN3 disease experienced a much faster diagnosis. Out of the five families that had a second child diagnosed with CLN2 or CLN3 disease, four children (*n* = 5) were diagnosed following the initial diagnosis of another sibling in their family. However, in one case (*n* = 5) the siblings were diagnosed at the same time. Three siblings were diagnosed prior to any symptom onset (*n* = 5) with the exception of two children (*n* = 5) that were already exhibiting symptoms. Genetic testing confirmed their diagnoses, triggered by an initial sibling in the family being diagnosed initially. The earlier diagnosis of siblings resulted in appropriate support services being initiated much earlier in the disease course and had a positive impact on the children’s ability to participate in education, including not having to attend countless diagnostic appointments that are disruptive to everyday life and school attendance. This is important because the initiation of adapted and special needs education for children with CLN3 disease contributes to improved learning conditions, better maintenance of skills and less frustration for affected individuals [36]. 

Non-specific initial symptoms and the rarity of CLN2 and CLN3 disease make it difficult for healthcare professionals to identify the disease. Opportunities to diagnose CLN2 and CLN3 disease earlier exist, and are discussed in this paragraph. The system should be designed to allow for early use of genetic or enzyme testing for NCLs. In CLN2 disease, sequencing a broad panel of mutations known to cause early onset epilepsies should be considered as first-line testing in all children with a first seizure under the age of 4 years [37]. Similarly, bilateral rapidly progressive vision loss, with or without childhood dementia and/or behavioural problems, in a very young child should immediately trigger testing, including CLN3 disease, to determine the cause [22, 38]. Before carrying out genetic testing, families should be informed about the purpose of testing and possible implications of the results, and give their informed consent alongside appropriate genetic counselling [37]. Batten disease is not included in the NHS newborn blood spot (NBS) screening programme that is offered to all babies at five days of age to test for nine serious but rare and treatable conditions [39]; CLN2 disease should be added immediately so it can be identified at the earliest possible opportunity, allowing for access to cerliponase alfa. For other rare conditions, such as Phenylketonuria (PKU), inclusion in the NBS screening programme has allowed for early identification, treatment and prevention of brain damage and severe long-term health problems for infants with the condition [39]. An additional issue is that standardised diagnostic pathways for CLN2 and CLN3 disease are currently lacking, resulting in children having to revisit doctors multiple times (over several years) with worsening symptoms, contributing to diagnostic delays.

Our study found that families take on enormous financial and care responsibilities. This is aligned with other studies that have found that parents of children living with rare chronic and complex diseases often experience significant health, psychosocial and economic burdens, alongside high unmet needs– regardless of the specific rare disease diagnosis, leading to a lower quality of life [40–42]. Similarly, adapting to the complex and changing needs of a child with CLN2 and CLN3 disease can be entirely overwhelming, both physically and mentally– leaving families feeling exhausted and drained. Many families temporarily took time off work or gave up work altogether to care for their child. Symptoms can change very quickly, and families feel they have to constantly be several steps ahead of the disease to ensure that supportive care is put in place in a timely manner. Families affected by CLN2 and CLN3 disease have to pre-emptively apply for services as their child’s symptoms are progressing in order to have timely access to the services they need; however, this often means they don’t meet the eligibility requirements at the time to access the needed social services. At the same time, uncovering what support families are entitled to is complicated and difficult to navigate, and social services are often not well suited to support rapidly progressive diseases. Consequently, families often lack the much-needed support from health and social care services. An example of best practice to support parental carers can be drawn from other countries. In Norway, parents are entitled to receive ‘attendance allowance’, which is a salary equivalent to their employment pay in order to care full time for a sick child, although the allowance cannot exceed NOK 638,394 annually (approximately GBP 46,000) [43]. 

These results are consistent with previous and recent studies revealing that the situation for families has not changed over the years. Schulz et al. (2020) studied family experiences in CLN2 disease in the UK and Germany and found that families had extensive care responsibilities, gave up work or significantly reduced their hours, and experienced financial burden from self-funding necessary home adaptations, care equipment and/or having to move to a more accessible property [27]. Another study by Krantz et al. (2022) investigating parental experiences of having a child with CLN3 disease in Sweden detailed the lack of understanding of NCLs in the health and social insurance systems, leading to a lack of timely access to the right support [28]. Moreover, a recent multi-country study (including the UK) by Schulz *et al. (2024)* found that 81% of parents were financially affected owing to increased expenses, reduced working hours, losing their job or stopping work altogether because of their child’s disease [30]. 

The impact on siblings of children with NCL has not been well researched to date but was revealed to be profound in this study. Despite the enormous effect on siblings, there are few resources available to them. Our findings align with a previous study by Malcolm et al. (2014), which found that siblings who take on an increased sense of responsibility and play a supportive role to their parents were often affected by the limitations constraining their family activities and by negative social attitudes towards disabilities [29]. 

A limitation of the study is that it focused on CLN2 and CLN3 diseases only, so it is unclear how the results translate to other NCL subtypes. A mix of voluntary response and purposive sampling was used as a method to recruit study participants, meaning all study participants had a prior connection with the BDFA. As a result, study findings may be more positively skewed due to the interviewees having support from a patient organisation, compared to families that are not connected with a patient support organisation.

Many of the themes uncovered in this study warrant further exploration in more specific studies, such as the emotional and mental impact on families with a child impacted by NCL (exhaustion and uncertainty); difficulties in communication with healthcare professionals (experiences and potential improvements); further quantifying financial and employment impact of caregiving on families in the UK. Also, the issue of genetic screening is complex, and proper exploration of the ethics, societal costs, economic scale, and practicality of genetic testing for rare and largely untreatable genetic conditions is a topic unto itself.

## Conclusion

To our knowledge, this is the largest recent UK study to provide an account of family experiences in CLN2 and CLN3 disease. We found the impact of CLN2 and CLN3 disease to be consuming and felt throughout all aspects of life. Families often had to fight for the health, education and social services they needed, starting from diagnosis. Families took on enormous care responsibilities that affected their employment and wellbeing. At the same time, they often had difficulty accessing the local health, education and social services they needed because of long waiting times, complicated eligibility requirements (e.g. home adaptations and other social services) or staff (e.g. social workers) turnover.

While the findings are limited to two types of disease, they call for more attention to NCLs as a whole. All forms of the disease are devastating. The progression of the most common types is well understood, and therefore family needs can be anticipated from the moment of diagnosis. More should be done to diagnose NCLs early, and ensure the cascade of support services are seamlessly provided throughout the care pathway and initiated for children and families at the earliest possible opportunity. This would allow children to maintain skills and quality of life for as long as possible and provide comprehensive support to the entire family.

## Electronic supplementary material

Below is the link to the electronic supplementary material.

Supplementary Material 1

## Data Availability

The data sets generated and/or analysed during the current study are not publicly available owing to patient and family confidentiality.
