# Supplementary material for: Evidence of the impact of CLN2 and CLN3 Batten disease on families in the United Kingdom
Source: Orphanet J Rare Dis. 2025 May 12;20:223. doi: 10.1186/s13023-025-03747-8 (PMC12067959; doi:10.1186/s13023-025-03747-8)
Supplement: Supplementary file 1 — Supplementary Material 1 [file 13023_2025_3747_MOESM1_ESM.docx]

# Appendix A. In-depth interview guide

To begin, I would like to get to know **your family** a bit better.

1. Can you start by describing who is in your family and tell me about them?
2. What does a typical day look like in your household?

I would like to ask about your experience **receiving a diagnosis** of Batten disease.

1. Can you describe when you first started noticing symptoms or difficulties in your child and what this was like for you?
2. How did you eventually receive a confirmed diagnosis of Batten disease for your child?
3. What challenges did you experience as a family, during this period?

I would like to ask about the **care and support** you receive for your child with Batten disease.

1. What has been the hardest aspect of caring for your child with Batten disease?
2. Do you receive adequate care and support to help manage Batten disease on a daily basis (e.g. social worker, occupational therapist etc)?
3. What aspects of support do you find most helpful? What about the least helpful?
4. Has your child been supported to continue their education?

I would like to understand the ways in which Batten disease has **impacted your family**.

1. What has been the most challenging or difficult aspect for you as a family?
2. What has been the impact on your other children, who do not have Batten disease?
3. Reflecting on everything that has been said today, is there anything else that you feel is important to share with us about your experience?

# Appendix B. Codebook for thematic analysis

| **Code** | **Description** |
| --- | --- |
| Awareness of Batten disease | Text describing awareness of Batten disease – including healthcare professional and family awareness of disease. |
| Batten disease community | Text describing interactions and thoughts families have had with the Batten disease community. This excludes mention of interactions with the community where families live, included in a separate code. |
| Caregiving | Text describing parental experiences on a day-to-day basis with caring for their child/children impacted by CLN2 or CLN3 disease. This includes the impact of caring on parental employment and impact of symptoms on children and parents. |
| Charities | Text describing support from local charities that families have received. |
| Community | Text that mentions interactions (positive or negative) of families affected by Batten disease with their immediate community. This excludes mention of family and friends, and the Batten disease community. |
| Diagnosis | ext describing the experience of families at diagnosis of Batten disease. This code includes testing (e.g. blood tests etc.), misdiagnosis and other related themes. It excludes text describing early signs and symptoms of Batten disease (covered by code ‘early symptoms’). Excludes text related to genetic counselling (covered by code ‘genetic counselling’). |
| Disease impact (patient) | This code represents the ways in which the disease (CLN2 or CLN3) interferes with the child’s daily life and ability to partake in activities. |
| Early symptoms | Any early signs and symptoms of children eventually diagnosed with CLN2 and CLN3 Batten disease (pre-diagnosis). |
| Education | Text describing children with Batten disease and their experience in obtaining quality education (including mainstream schools and/or special schools). It excludes unaffected siblings. |
| Emotional impact (parents) | Text describing the emotional impact on parents of having a child with Batten disease. |
| Employment (parents) | Text describing the impact of Batten disease on parents’ employment status. |
| Family impact | Text describing the impact (emotional, spiritual etc.) of having Batten disease on immediate family, and how the disease affects the family unit. |
| Financial impact | Financial burden of Batten disease on families. It excludes any public funding that families may have received for home adaptations (covered by code ‘home environment’) or community support (covered by code ‘community’). |
| Genetic counselling | Text including advice, information and counselling given to families regarding the genetics of Batten disease and implications for family unit. |
| Home environment | Text describing the suitability of the home environment for a child with Batten disease, with physical disability and complex needs, including home adaptations. |
| Relationships (friends and family) | Text describing the impact of Batten disease on relationships with extended family and friends. Excludes impact of Batten disease on immediate family, (covered by code ‘family impact’). |
| Sibling impact | This code includes text detailing the impact of unaffected siblings. |
| Support services | Support provided to the family (including social, emotional, allied healthcare professionals, charities, financial) to help manage their child/children with CLN2 or CLN3. Excludes community, family/friend and school support (covered in separate codes). |
| Treatment and management | This code includes text excerpts that mention treatment that children with Batten disease (CLN2 and CLN3) had. It includes symptom management (seizures, behavioural etc.), cerliponase alfa treatment, care teams and medications. |
